# Prevalence and factors of physical punishment and psychological aggression toward children under five in Mongolia: an analysis of the 2018 Social Indicator Survey

**DOI:** 10.1080/16549716.2024.2397838

**Published:** 2024-09-10

**Authors:** Anujin Enkhbat, Seungman Cha, Ermias Tadesse Beyene, Yan Jin

**Affiliations:** a Department of Global Development and Entrepreneurship, Graduate School of Global Development and Entrepreneurship, Handong Global University, Pohang, South Korea; b Department of Human Ecology and Technology, Graduate School of Advanced Convergence, Handong Global University, Pohang, South Korea; c Department of Microbiology, Dongguk University College of Medicine, Gyeongju, South Korea

**Keywords:** Physical punishment, psychological aggression, Under-five children, Mongolia, social indicator survey

## Abstract

**Background:**

The global prevalence of violence against children is alarmingly high, with millions facing violent discipline and physical punishment. In Mongolia, domestic violence-related criminal offenses have sharply increased, with a 46.92% surge in the first quarter of 2020 compared to 2019.

**Objective:**

This study aimed to estimate the prevalence of and identify factors associated with physical punishment and/or psychological aggression experienced by children under 5 years old from their caregivers.

**Methods:**

We used data from the nationally representative 2018 MICS6 dataset. To examine the association between independent and dependent variables, we used multilevel Poisson regression because it provides a better estimate and is more interpretable when the prevalence is relatively high.

**Results:**

The prevalence of psychological aggression was reported at 32.3% and physical punishment at 31.6%, including severe forms. Nonviolent techniques were common, with 77.5% exclusively using nonviolent discipline. Psychological aggression was more likely to occur in older children (3 and 4 years old) and in households with Buddhist heads. Additionally, 3-year-olds are more likely to experience physical punishment compared to 2-year-olds.

**Conclusion:**

These findings underscore the need for targeted policy interventions, including age-sensitive parental education programs and religious and cultural sensitivity measures. Comprehensive educational and awareness programs are essential to foster a culture of nonviolence across all educational levels, highlighting the need for context-specific policies to safeguard the well-being of children in Mongolia.

## Paper Context

**Main finding:**

The study highlights concerning rates of physical punishment and psychological aggression toward children under five in Mongolia.

**Added Knowledge:**

The study findings contribute novel insights into the intricate relationship between sociocultural factors and disciplinary practices, emphasizing the influence of religious affiliations and maternal education on child-rearing approaches.

**Global health impact for policy and action:**

Urgent policy interventions are warranted to address violence against children, with an emphasis on culturally sensitive parental education programs and comprehensive awareness campaigns.

## Background

Historically, instances of child maltreatment and domestic violence remained largely obscured within the judicial system unless they were notably severe [1]. In the late 1890s, Freud suggested that many psychological disorders in women could be traced back to childhood or adolescence, predominantly perpetrated by family members or close relatives [1]. However, his contemporaries dismissed these ideas as mere ‘fantasies.’ This perspective was reinforced by the prevailing belief that children were not dependable narrators and that child sexual abuse was rare [1].

A shift began in the 1960s and 1970s when the magnitude of unreported domestic and child abuse was recognized [1]. This led to the creation of support systems for victims of intrafamilial violence and spurred research into understanding the impact and extent of this abuse [1]. By the 1980s, many legal systems had begun reforms to better address the needs of women and children affected by familial abuse, as highlighted by Bala in 2008 [1].

The prevalence of violence against children is alarmingly high globally [2]. In 2017, nearly 300 million children aged 2–4 endured violent discipline and 250 million faced physical punishment [2]. One out of every four children under five lives with a mother who has been a victim of intimate partner violence [2]. A study from 30 countries revealed that 60% of children between 12 and 23 months experienced physical discipline and almost 50% faced verbal abuse [2]. Despite over one billion caregivers believing in physical punishment as a necessary method, only 59 nations have fully prohibited it in domestic settings [2]. This left more than 600 million children without legal protection from such violence in 2017, according to UNICEF [2].

The evolution of child rights in Mongolia has been significant, transitioning from communism to democracy from 1921 to 1990 [3]. In 1996, the Law on the Protection of the Rights of the Child was enacted and later revised in 2016 as the Child Protection Law, with the government endorsing the National Programme of Action on Child Protection and Development in 2017 [3]. This commitment is reflected in the 2018 Multiple Indicator Cluster Survey, which addressed child discipline issues [4]. However, recent reports from Save the Children show a concerning rise in domestic violence-related offenses, particularly against children [5]. In the first quarter of 2020, incidents surged by 46.92% compared to 2019, with notable increases in physical and psychological abuse, as well as neglect [5].

The UNICEF report evaluated Mongolia’s child welfare using 44 Sustainable Development Goal (SDG) indicators, grouped into five areas: survive and thrive, learning, protection, the environment, and fair chance [6]. In Mongolia, the protection area requires significant effort to meet targets or improve monitoring, as highlighted in the report. Data collection challenges were faced for four out of nine indicators in this area, creating uncertainty about their current status [6]. While two indicators, specifically 12% of the women (aged 20–24 years) married or were in a union before the age of 18 and 49% of the children (aged 1–14 years) experienced physical punishment or psychological aggression from caregivers, have been monitored, the available trend data are insufficient to draw any definitive conclusions [6]. However, one indicator has already achieved its target, indicating progress. Nevertheless, more effort is necessary, especially in reaching SDG 16.2.1, suggesting that further action is needed to accomplish SDGs goals [6].

The topic of physical punishment and psychological aggression has not been thoroughly investigated in many developing countries, despite it being a common and critical public health issue [7,8]. A previous multicountry study asserted that the highest prevalence of all forms of child abuse was in African countries [7]. Countries such as Kazakhstan, Kyrgyzstan, Mongolia, and Turkmenistan in Central Asia demonstrate relatively low percentages of children experiencing physical abuse (1–6.5%) [9]. On the other hand, Chad, Congo-Kinshasa, and Niger showed higher percentages of physical abuse targeting children under 5 years of age, reaching up to 43.2%, 42.1%, and 38.4%, respectively, in 2011–2015 (0–59 months) [9]. Child abuse often occurs during punishment and is linked to mental health issues such as depression, alcohol abuse, and suicide [10]. A systematic review found that child abuse increases the risk of depressive disorders across all income levels [11]. Bullying among youth is often connected to domestic abuse [12].

Over the past decade, studies have consistently highlighted the trend of violent disciplinary practices against Mongolian children [13]. The 2018 National Statistical Office of Mongolia Survey, which sampled 15,168 children aged 1–14, found that nearly half of the children (49.1%) had recently faced violent discipline, with 5.2% enduring severe physical punishment [14]. This is in line with the findings from the 2013 Multiple Indicator Cluster Survey (MICS), which documented a similar 49% prevalence rate among children of the same age bracket [15]. The 2013 MICS survey indicated that younger children (aged 3–4 years) encountered the most frequent physical punishment (44%), while older children aged 10–14 years faced it less frequently (16%) [15]. Furthermore, a 2012 study focusing on the Nalaikh District revealed that 42% of the children aged 2–14 years were victims of psychological or physical punishment in the preceding month [16]. The 2010 MICS Survey indicated that 46% of the children aged 2–14 years had experienced violent disciplinary methods at home [17].

Although the literature provides a strong quantitative understanding of child maltreatment in Mongolia, there is still a gap. Previous studies have mainly focused on children aged 1–14 as a whole, but a more detailed age-specific analysis is needed to gain deeper insights into this specific age group. Additionally, there is a lack of documentation on individual and regional characteristics that may influence the factors contributing to physical punishment and psychological aggression toward children in Mongolia. This study sought to address this gap.

We analyzed the prevalence and associated factors of physical punishment and/or psychological aggression toward children under the age of five by their caregivers. To the best of our knowledge, this is the first study to address maltreatment of children under five in Mongolia.

## Materials and method

### Study design and setting

This study utilized secondary data from the UNICEF Multiple Indicator Cluster Surveys (MICS), for which we obtained permission on 2 November 2022. The sixth round of the MICS, adopted by Mongolia and renamed the Social Indicator Sample Survey (SISS), was conducted in 2018 by the National Statistical Office (NSO) of Mongolia in collaboration with UNICEF and the United Nations Population Fund (UNFPA). The SISS collected data on health, education, development, protection, well-being, and the rights of children and women using structured survey questionnaires. The survey had a response rate of 97.3%. Detailed sampling techniques and additional information are published elsewhere [14]. We have attempted to adhere to the standard guidelines for cross-sectional studies in reference to the Strengthening the Reporting of Observational Studies in Epidemiology (STROBE) checklist from the Enhancing the Quality and Transparency of Health Research (EQUATOR) (Appendix 1).

The 2018, MICS sample used stratified two-stage cluster sampling, dividing 13 province districts into urban and rural areas. The sampling framework was based on the 2017 Population and Household Registry. The first stage involved selecting enumeration areas (EAs) with a probability proportional to their size. After household listing in the sampled EAs in August and September 2018, a systematic sample of 25 households was selected from each PSU. A countrywide sample of 14,500 households was selected, with one child randomly chosen for interviews in each household.

Our study focused on children under 5 years old, sampling 6,269 children from selected households to investigate social indicators for this age group. The under-5 questionnaire was specifically administered to mothers or primary caretakers of one randomly selected child under five living in the household. In terms of data collection, the survey utilized Computer-Assisted Personal Interviewing (CAPI). Data was collected using tablet computers running the Windows 10 operating system. The field operations employed a Bluetooth application that facilitated the transfer of assignments and completed questionnaires between supervisor and interviewer tablets.

### Study model

Examining factors contributing to the prevalence of child maltreatment involved investigating two dependent variables: children experiencing physical punishment (moderate and severe) and psychological aggression. The assessment utilized a modified version of the ‘Parent-Child Conflict Tactics Scale’ (PCCTS) [18] to evaluate 11 child disciplinary behaviors (Table 1) exhibited by household members with children under five in the survey conducted by the MICS in the month preceding the survey [14]. The MICS questionnaire included three questions on nonviolent disciplinary practices, two on psychological aggression, and six on physical punishment. Respondents indicated whether they or any family member used any disciplinary method on a child in the past month. Data analysis consolidated individual disciplinary practices into three primary scales: nonviolent discipline, psychological aggression, and physical punishment. Responses were categorized into these scales based on reported practices. Table 1 lists the practices included in each subscale.

**Table 1.** Basic characteristics of the children.

Variable		N	(%)
Sex of child	Male	1978	51.98
	Female	1827	48.02
Child’s age	2	1228	32.27
	3	1278	33.59
	4	1299	34.14
Area	Capital city	840	22.08
	Aimag center	1083	28.46
	Soum center	835	21.94
	Rural area	1047	27.52
Region	Western	1010	26.54
	Khangai	776	20.39
	Central	627	16.48
	Eastern	552	14.51
	Ulaanbaatar	840	22.08
Religion of household-head	No religion	1577	41.45
	Buddhist	1692	44.47
	Muslim	363	9.54
	Others	157	4.13
Ethnicity of household-head	Khalkh	2886	75.85
	Kazakh	402	10.57
	Others	503	13.22
Mother’s education level	Pre-primary or none	213	5.60
	Primary	250	6.57
	Basic (lower secondary)	598	15.72
	Upper secondary	767	20.16
	Vocational	497	13.06
	College, university	1480	38.90
Wealth index quintile	Poorest	1097	28.83
	Second	959	25.20
	Middle	709	18.63
	Fourth	602	15.82
	Richest	438	11.51
Child functional difficulties	Has difficulties	87	2.29
	No difficulties	3707	97.42
Ever attended school or Early childhood program	Yes	3733	98.11
	No	72	1.89

*ECP: Early Childhood Program.

*Children aged 1 year or below were excluded, as functional difficulties were only collected from children aged 2–5 years.

In this study, the independent variables were selected based on relevant literature, including reports on the specific context of Mongolia and the availability of variables within the dataset [19–21]. The independent variables were classified into individuals (child and household) and regional characteristics of children under five.

### Study subjects

**Figure 1.**
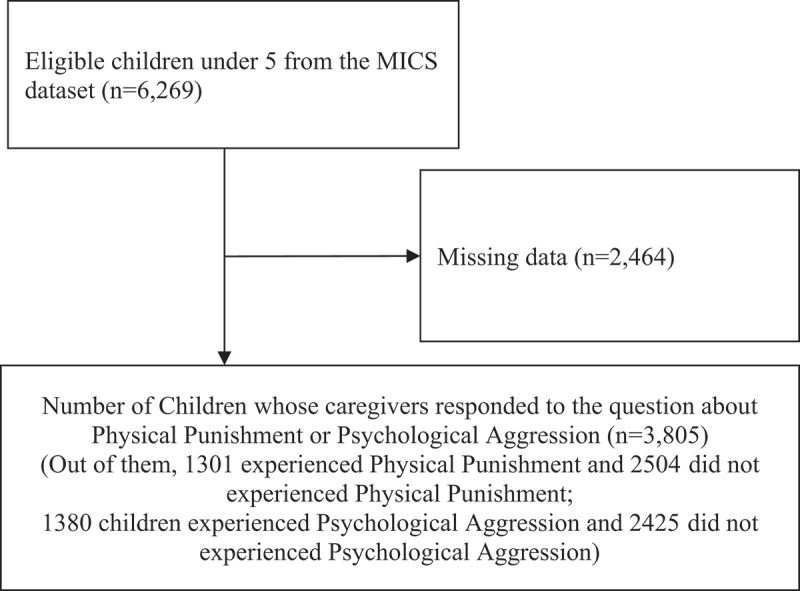
Selection of study participants.

The subjects of this study focused on children aged 2–4 years in Mongolia, totaling 6,269 from selected households in the MICS dataset. Children aged 0–1 were excluded because functional difficulties data were only collected for those aged 2–4. Respondents who provided ‘no answer’ (2,464) regarding physical punishment and psychological aggression in the month before the survey were excluded from the study. The number of children under five who responded to the survey was 3,805 for physical punishment and 3,805 for psychological aggression. The final study subjects consisted of 1,301 children who experienced physical punishment and 1,380 children who experienced psychological aggression, out of the initial 6,269 (Figure 1).

### Analysis methods

The MICS dataset employs a two-stage stratified cluster sampling design, in which primary sampling units (PSUs) represented by enumeration areas (EAs) are initially chosen, followed by the selection of households within these clusters [14]. This creates a natural hierarchical structure with households nested within EAs, forming the basis for employing multilevel modeling to analyze the clustered or nested data structure. The data collected through the MICS exhibited a hierarchical structure, with households nested within EAs and potentially nested within regions [14]. Given the focus on physical punishment and psychological aggression toward children aged 1–4 years, shared characteristics may exist within these clusters (EAs).

### Study variables

#### Dependent variables

The dependent variables in this study were physical punishment and psychological aggression in children under 5 years of age in Mongolia within the month prior to the survey (Appendix 2). The MICS Dataset was used for assessment. Moderate physical punishment was determined by asking caregivers about actions such as shaking a child, spanking, slapping a child’s bottom, or hitting the hand, arm, or leg. A ‘yes’ response from caregivers indicated that the child experienced moderate physical punishment, with data coded as ‘1’ for those who did and ‘0’ for those who did not. Severe physical punishment was identified by asking caregivers about actions such as hitting the face, head, or ears, using objects, or beating the child intensely in the last month. Similarly, ‘yes’ responses signified severe physical punishment, coded as ‘1’ for those who experienced it, and ‘0’ for those who did not. The assessment of psychological aggression involved asking parents about shouting, yelling, or using derogatory language. Scores from these questions were combined to create a binary outcome: ‘1’ for children experiencing any form of psychological aggression and ‘0’ for those who did not encounter such aggression, utilizing the MICS dataset. Analyzing the variables associated with child maltreatment, this study excluded children who answered ‘don’t know’ and ‘missing values’ from the study, and the dependent variables were classified only as ‘yes’ or ‘no.’

We used a simple ‘Yes’ response to indicate physical or psychological abuse, while other studies have employed more detailed measures that take into account the frequency and context of abusive acts [22,23]. In the discussion section, we will explore these methodological differences to offer a more comprehensive understanding of our approach and its implications.

#### Independent variables

The independent variables were classified into child, household, and regional characteristics of children under 5 years of age.

First, the child characteristics encompassed the child’s sex, age in years, and functional difficulties [20]. The children were categorized into male and female, and their age was divided into four groups: 1, 2, 3, and 4 years. Children aged 0–1 years were excluded from the MICS dataset, as functional difficulties are only collected for children aged 2–4 years in the MICS dataset. The questionnaire for children’s functional domains included aspects such as vision, hearing, mobility, fine motor skills, communication, learning, play, and behavioral control.

Second, the household characteristics were the mother’s education level, religion and ethnicity of the household-head, wealth index quintile, and whether the child has attended a school or early childhood program (ECP) [21]. The mother’s education level was divided based on the Mongolian education system. These categories include pre-primary or none, primary, basic (lower secondary), upper secondary, vocational and college, and university education. Religion was transformed into numerical labels, with ‘1’ representing no religion, ‘2’ denoting Buddhist, and ‘3’ indicating Muslim. Additionally, ‘6’ was employed to designate other religions. For ethnicity representation, ‘1’ denoted the Khalkh ethnicity, ‘2’ represented the Kazakh ethnicity, and ‘6’ was used to indicate other ethnicities such as Burvud, Buriat, Bariganga, and Bayad. The 2018 MICS calculates national-level wealth quintiles by categorizing households into five equal groups: the poorest, poor, middle, rich, and the richest. Attendance of school or ECP by the child was classified as ‘yes’ or ‘no.’

Third, the regional characteristics were classified by distinguishing between urban and rural areas, considering regional aspects. In the Mongolian context, location categories were further detailed to include the capital city, Aimag center, Soum center, and rural areas. The region was divided into five distinct regions: Eastern, Western, Central, Khangai, and Ulaanbaatar.

### Statistical analysis

The data were analyzed using multilevel Poisson regression because it provides a better estimate and is more interpretable when the prevalence is relatively high [22,23]. Child maltreatment is a complex phenomenon influenced by a myriad of factors at individual and regional levels [24]. In this context, employing multilevel models has emerged as a promising research strategy, offering the potential to unravel the intricate interactions between individual and regional factors that contribute to child maltreatment [24]. Therefore, to identify factors affecting the prevalence of psychological aggression and physical punishment, we employed a multilevel Poisson regression analysis. We used Poisson regression because the prevalence of physical punishment and psychological aggression were relatively high, and prevalence ratio, the result of Poisson regression, was easy to understand compared to odds ratio.

Prior to multilevel Poisson regression analysis, a bivariate Poisson regression analysis is conducted in order to determine the association between the dependent and the potential independent variables. As a result, in the final multilevel Poisson regression model categorical variables which have a *p* value of <0.25. We declare the statistical significance of all variables which have *p* value of <0.05 with a prevalence ratio (PR). Data analyses were performed using RStudio (version 2022.07.2 ‘Spotted Wakerobin;’ RStudio, PBC, Boston, MA, USA; release 6 September 2022) for macOS.

A smaller number of clusters in multilevel models reduce the statistical power to detect a non-null random effects variance. We used a likelihood test to assess whether clustering exerts an effect on the outcome based on existing literature due to the small number of clusters in our study [24].

Three models were used to examine the prevalence of disciplinary acts performed on a child, sequentially entering the individual and regional characteristics. Model I incorporates the individual characteristics (child and household variables) such as the child’s sex, age, functional difficulties, mother’s education level, religion and ethnicity of the household-head, wealth index quintile, and whether the child has attended school or an early childhood program (ECP). Model II included regional characteristic variables, introducing regions and areas. Finally, Model III is a comprehensive model that includes both the individual and regional characteristic variables. The model used for the analysis is as follows: -

$$\mathrm{Log}\left(P_{\mathrm{ij}}\right)\;=B_{00}+B_{10}*x_{\mathrm{ij}}+B_{01}*X_{j}+U_{0j}+e_{\mathrm{ij}}$$

Where P_ij_ is the probability of the outcome of interest for child i in region j; *B*s are the fixed coefficients (Prevalence Ratio); x and X represent the individual- and regional-level independent variables, respectively; *U* indicates the random effects for the jth region; and ε shows the unmeasured factors that may influence the primary outcome of interest.

The deviance, defined as −2 × LN (likelihood), indicates the model fit of the data, where LN represents the natural logarithm and likelihood is the value of the likelihood function at convergence. The lower the deviance, the better the model fits. In this study, all the models we compared were nested, meaning a more general model can derive a more specific model by removing some parameters. In the two nested models, the difference of the deviances follows a chi-square distribution. We performed the likelihood ratio test to explore the difference in the deviance between the two models.

## Results

### Descriptive characteristics of the sample

Table 1 presents the basic characteristics of the 3805 children aged 2–4 in Mongolia. Most were male (51%), and the Western region had the highest representation (26%). The majority of respondents belonged to the Khalkh ethnic group (75%), and 38% of mothers had completed college or university education.

### Prevalence of physical punishment and psychological aggression

The prevalence of child disciplinary methods in Mongolia for children aged 2–4 is detailed in Table 2. Nonviolent techniques were common, with 77.5% exclusively using nonviolent discipline. However, psychological aggression, reported at 32.3%, and physical punishment, at 31.6%, including severe forms, were significant.

**Table 2.** Prevalence of child disciplinary methods in the past month in children aged 2–4 years, Mongolia, 2018.

Disciplinary Method acts	% (95% Confidence Interval)
Non-violent discipline	
1. ‘Explained why (name)’s behavior was wrong.’	71.5 (70.3, 72.8)
2. ‘Took away privileges, forbade something (name) liked, or did not allow (him/her) to leave the house.’	21.9 (20.7, 23.0)
3. ‘Gave (him/her) something else to do.’	22.7 (21.6, 23.9)
Non-violent discipline only	77.5 (76.3, 78.7)
Psychological aggression (items 4 – 5)	32.3 (31.0, 33.6)
4. ‘Shouted, yelled at, or screamed at (him/her).’	31.2 (29.9, 32.5)
5. ‘Called (him/her) dumb, lazy, or other names like that.’	4.0 (3.5, 4.6)
Physical punishment (items 6 – 11)	31.6 (30.3, 32.9)
6. ‘Spanked, hit, or slapped (him/her) on the bottom with bare hand.’	22.6 (21.4, 23.8)
7. ‘Hit or slapped (him/her) on the hand, arm, or leg.’	9.0 (8.2, 9.8)
8. Shook (him/her).”	8.1 (7.3, 8.9)
9. ‘Hit (him/her) on the bottom or elsewhere on the body with something like a belt, hairbrush, stick, or other hard objects.’	2.1 (1.7, 2.5)
10. ‘Hit or slapped (him/her) on the face, head, or ears’.	2.8 (2.4, 3.3)
11. ‘Beat (him/her) up that is hit (him/her) over and over as hard as one could.’	1.5 (1.2, 1.9)
Severe physical punishment (items 10 – 11)	4.1 (3.5, 4.7)

In Mongolia in 2018, physical punishment and psychological aggression prevalence varied by demographic and socioeconomic factors (Table 3). Male children experienced higher physical punishment rates (36.8%) than females (31.36%, *p* < 0.001), with older children (3–4 years: 36.38%, 34.79%) more affected than 2-year-olds (31.27%, *p* = 0.02). Geographically, the capital city had the highest prevalence (37.97%), while the Western region had the lowest (30.69%). Physical punishment rates differed across religions, with Buddhists at 39.59% and Muslims at 29.75%. Mother’s education and wealth quintiles also showed variations. No statistically valid link was found between psychological aggression and gender difference or variations across areas, wealth quintiles, or mother’s education level.

**Table 3.** Prevalence of physical punishment and psychological aggression by demographic and socio-economic factors in Mongolia, 2018.

Variables		Physical			Psychological		
		Punishment			Aggression		
		No (%)	Yes (%)	P-value	No (%)	Yes (%)	P-value
Sex of child	Male	63.19	36.8	<0.001	62.63	37.36	0.15
	Female	68.63	31.36		64.91	35.08	
Child’s age	2	68.72	31.27	0.02	71.17	28.82	<0.001
	3	63.61	36.38		61.73	38.26	
	4	65.2	34.79		58.66	41.33	
Area	Capital city	62.02	37.97	0.02	62.97	37.02	0.21
	Aimag center	68.05	31.94		61.68	38.31	
	Soum center	68.02	31.97		66.1	33.89	
	Rural area	64.75	35.24		64.56	35.43	
Region	Western	69.306	30.69	0.02	63.36	36.63	0.14
	Khangai	64.56	35.43		62.62	37.37	
	Central	66.02	33.97		62.36	37.63	
	Eastern	66.66	33.33		68.65	31.34	
	Ulaanbaatar	62.02	37.97		62.97	37.02	
Religion of household-head	No religion	66.83	33.16	0.10	67.85	32.14	<0.001
	Buddhist	64.47	35.52		60.4	39.59	
	Muslim	70.24	29.75		63.36	36.63	
	Others	59.87	40.12		59.87	40.12	
Ethnicity of household-head	Khalkh	65.55	34.44	0.01	63.51	36.48	0.90
	Kazakh	72.38	27.61		64.67	35.32	
	Others	61.82	38.17		64.01	35.98	
Mother’s education level	Pre-primary or none	72.76	27.23	0.02	67.13	32.86	0.32
	Primary	64	36		63.2	36.8	
	Basic (lower secondary)	70.06	29.93		67.39	32.6	
	Upper secondary	65.71	34.28		62.58	37.41	
	Vocational	62.77	37.22		62.17	37.82	
	College, university	64.45	35.54		62.97	37.02	
Wealth index quintile	Poorest	65.45	34.54	0.01	66.08	33.91	0.23
	Second	68.3	31.69		64.23	35.76	
	Middle	67.98	32.01		62.9	37.09	
	Fourth	65.28	34.71		61.62	38.37	
	Richest	58.44	41.55		60.95	39.04	
Child functional difficulties	Has difficulties	68.96	31.03	0.81	64.36	35.63	0.99
	No difficulties	65.74	34.25		63.71	36.28	
Ever attended school or Early childhood program	Yes	65.63	34.36	0.12	63.62	36.37	0.37
	No	75	25		69.44	30.55	

*ECP: Early Childhood Program; *p*-values obtained using chi-square tests.

The mixed-effect multilevel Poisson regression analyses in Tables 4 and 5 demonstrated significant correlations between various individual and regional-level factors and the prevalence of psychological aggression and physical punishment toward children under five in Mongolia.

**Table 4.** Multilevel mixed-effect Poisson regression analysis of individual and regional-level factors associated with the prevalence of psychological aggression toward children under five in Mongolia, 2018.

Characteristics fixed effect		Null Model	Model I aPR^a^ [95% CI]^b^		Model II aPR [95% CI]		Model III aPR [95% CI]		
Sex of child	Male (ref)		1					1	
	Female		0.94 (0.85, 1.05)	***				0.94 (0.85, 1.05)	
Child’s age	2(ref)		1					1	
	3		1.32 (1.15, 1.52)	***				1.32 (1.15, 1.52)	***
	4		1.43 (1.25, 1.64)					1.43 (1.25, 1.63)	***
Child functional difficulties	Has difficulties(ref)		1					1	
	No difficulties		1.04 (0.73, 1.49)					1.03 (0.72, 1.47)	
Mother’s education level	Pre-primary or none (ref)		1					1	
	Primary		1.06 (0.75, 1.50)					1.07 (0.75, 1.52)	
	Basic (lower secondary)		0.93 (0.67, 1.28)					0.95 (0.69, 1.31)	
	Upper secondary		1.06 (0.77, 1.46)					1.08 (0.79, 1.49)	
	Vocational		1.08 (0.78, 1.49)					1.10 (0.79, 1.52)	
	College, university		1.00 (0.72, 1.37)	***				1.02 (0.74, 1.40)	
Religion of household-head	No religion (ref)		1					1	
	Buddhist		1.22 (1.09, 1.37)					1.21 (1.08, 1.36)	**
	Muslim		1.30 (0.83, 2.03)					1.25 (0.80, 1.95)	
	Others		1.25 (0.96, 1.63)					1.26 (0.97, 1.64)	
Ethnicity of household-head	Khalkh (ref)		1					1	
	Kazakh		0.88 (0.57, 1.36)					0.87 (0.56, 1.35)	
	Others		1.01 (0.86, 1.19)					1.02 (0.87, 1.20)	
Wealth index quintile	Poorest (ref)		1					1	
	Second		1.04 (0.89, 1.21)					1.03 (0.86, 1.24)	
	Middle		1.08 (0.91, 1.29)					1.07 (0.88, 1.31)	
	Fourth		1.12 (0.93, 1.35)					1.12 (0.90, 1.39)	
	Richest		1.14 (0.92, 1.40)					1.11 (0.86, 1.44)	
Ever attended school or Early childhood program	Yes(ref)		1					1	
	No		1.15 (0.69, 1.91)					1.12 (0.68, 1.86)	
Region	Western(ref)					1		1	
	Khangai					1.01 (0.86, 1.18)		0.97 (0.82, 1.16)	
	Central					1.02 (0.86, 2.18)		1.00 (0.83, 1.20)	
	Eastern					0.85 (8.86, 0.18)		0.86 (0.70, 1.04)	
	Ulaanbaatar					1.02 (0.86, 2.18)		0.94 (0.75, 1.19)	
Model fitness test results	Deviance	5559.28	5507.38			5551.66		5502.94	
	p-value for the likelihood ratio test		*p* < 0.01 (compared to Null model) *p* < 0.01 (compared to Model II)			*p* < 0.05 (compared to Null model)		*p* < 0.01 (compared to Null model) *p* < 0.05 (Compared to Model I)	

^a^adjusted prevalence ratio.

^b^95% confidence interval.

*Adjusted Prevalence Ratio; Significant. codes: ‘***’*p* < 0.001, ‘**’*p* < 0.01, ‘*’*p* < 0.05, ref=reference category.

**Table 5.** Multilevel mixed-effect Poisson regression analysis of individual and regional-level factors associated with the prevalence of physical punishment toward children under-five in Mongolia, 2018.

Characteristics fixed effect		Null Model	Model I aPR^b^ [95% CI] ^b^	Model II aPR [95% CI]			Model III aPR [ 95% CI]	
Sex of child	Male (ref)		1				1	
	Female		0.85 (0.76, 0.95)	**			0.85 (0.76, 0.95)	**
Child’s age	2(ref)		1				1	
	3		1.17 (1.02, 1.34)	*			1.16 (1.02, 1.33)	*
	4		1.11 (0.97, 1.27)				1.11 (0.97, 1.28)	
Child functional difficulties	Has difficulties(ref)		1				1	
	No difficulties		0.96 (0.65, 1.40)				0.97 (0.66, 1.42)	
Mother’s education level	Pre-primary or none (ref)		1				1	
	Primary		1.32 (0.91, 1.92)				1.33 (0.91, 1.93)	
	Basic (lower secondary)		1.10 (0.78, 1.56)				1.10 (0.78, 1.57)	
	Upper secondary		1.26 (0.90, 1.78)				1.27 (0.90, 1.79)	
	Vocational		1.37 (0.97, 1.95)				1.38 (0.97, 1.96)	
	College, university		1.27 (0.90, 1.79)				1.28 (0.91, 1.82)	
Religion of household-head	No religion (ref)		1				1	
	Buddhist		1.06 (0.94, 1.19)				1.05 (0.94, 1.19)	
	Muslim		1.50 (0.93, 2.43)				1.55 (0.95, 2.51)	
	Others		1.20 (0.92, 1.56)				1.17 (0.90, 1.53)	
Ethnicity of household-head	Khalkh (ref)		1				1	
	Kazakh		0.60 (0.37, 0.96)	*			0.61 (0.38, 0.98)	*
	Others		1.10 (0.94, 1.29)				1.09 (0.93, 1.28)	
Wealth index quintile	Poorest (ref)		1				1	
	Second		0.89 (0.76, 1.04)				0.92 (0.77, 1.11)	
	Middle		0.89 (0.74, 1.06)				0.91 (0.74, 1.12)	
	Fourth		0.95 (0.79, 1.16)				0.99 (0.79, 1.24)	
	Richest		1.12 (0.91, 1.38)				1.14 (0.87, 1.48)	
Ever attended school or Early childhood program	Yes(ref)		1				1	
	No		1.11 (0.63, 1.94)				1.10 (0.63, 1.93)	
Region	Western(ref)				1		1	
	Khangai				1.15 (0.98, 1.35)		1.09 (0.91, 1.31)	
	Central				1.12 (0.94, 1.34)		1.06 (0.88, 1.29)	
	Eastern				1.08 (0.90, 1.30)		1.03 (0.84, 1.26)	
	Ulaanbaatar				1.16 (0.98, 1.39)		1.07 (0.84, 1.36)	
Model fitness test results	Deviance	5559.28	5507.38		5551.66		5502.94	
	p-value for the likelihood ratio test		*p* < 0.01 (compared to Null model) *p* < 0.01 (compared to Model II)		*p* < 0.05 (compared to Null model)		*p* < 0.01 (compared to Null model) *p* < 0.05 (Compared to Model I)	

^a^adjusted prevalence ratio.

^b^95% confidence interval.

*Adjusted Prevalence Ratio; Significant. codes: ‘***’*p* < 0.001, ‘**’*p* < 0.01, ‘*’*p* < 0.05, ref=reference category.

Children aged 3 are 32% more likely to experience psychological aggression compared to 2-year-olds, with an adjusted prevalence ratio (aPR) of 1.32 (95% Confidence Interval: 1.15, 1.52). Children aged 4 are 43% more likely to experience psychological aggression compared to 2-year olds with an aPR of 1.43 (95% CI: 1.25, 1.63). The religion of the household head also influences psychological aggression. Children from Buddhist households are 21% more likely to experience it compared to those from households with no religion (aPR 1.21, 95% CI: 1.08, 1.36). Children from Muslim households (aPR 1.25, 95% CI: 0.80, 1.95) and other religions (APR 1.26, 95% CI: 0.97, 1.64) also have a higher tendency of experiencing psychological aggression than those with no religion, although these differences are not statistically significant.

For physical punishment, age is a significant factor. Three-year-olds are 16% more likely to experience physical punishment compared to 2-year-olds (aPR 1.16, 95% CI: 1.02, 1.33). Female children, compared to male children, exhibit a 15% lower prevalence of physical punishment under the age of five (aPR = 0.85, 95% CI: 0.76, 0.95). Children from Kazakh households are 39% less likely to experience physical punishment compared to those from Khalkh households (aPR of 0.61, 95% CI: 0.38, 0.98)

Model III has the lowest deviance compared to the preceding models from both physical punishment and psychological aggression models, and as a result, it is the best fitting model for analysis.

## Discussion

This study indicates that in the month prior to the survey, 32.3% of children under the age of five in Mongolia had encountered physical punishment, and 31.6% had experienced psychological aggression.

The two child-disciplining methods exhibited significant associations with the child’s age, the sex of the child, religion of household-head, and ethnicity of household head.

Children aged 3 and 4 are more prone to psychological aggression and physical punishment than 2-year-olds, likely due to the challenging behaviors typical of toddlers. Toddlers’ exploration and boundary-testing can lead to parental stress and frustration, prompting resorting to physical discipline [25,26]. Parents may perceive toddler behaviors as intentional misbehaviors, contributing to increased use of physical punishment [27,28].

This study discovered a gender-based difference in the prevalence of physical punishment, showing that girls are less likely to experience psychological aggression and physical punishment than boys. The majority of these studies also found that physical punishment is linked to increased problem behavior in both genders, although the strength of this association may vary [29]. Research consistently indicates that boys are more likely to endure severe physical punishment compared to girls, highlighting a greater risk for harsh discipline among male children [30,31].

The higher likelihood of psychological aggression toward children under five in families with Buddhist household heads prompts a deeper exploration of the relationship between religious affiliations and caregiving practices. This trend raises questions about the potential influence of cultural teachings in Buddhist backgrounds.

The lack of peer-reviewed papers specifically addressing psychological aggression in Mongolia led us to explore alternative sources. A 2021 UNICEF report highlighted educational disparities within Mongolian monasteries, where children face inequalities in accessing fundamental education and health and hygiene education compared to regular schools [29]. These disparities contribute to the complex dynamics observed in our study regarding psychological aggression towards young children.

Educational disparities among Buddhist monasteries may have contributed to the observed patterns of psychological aggression, emphasizing the unique challenges faced by children in these settings. Additionally, the influential role of monks in these monasteries, serving as religious leaders and mentors for families, plays a significant role in shaping cultural and educational practices.

This underscores the need for a comprehensive understanding of the sociocultural context and educational disparities within different settings to effectively address and mitigate psychological aggression toward children in Mongolia.

The study also found that children from Kazakh households are less likely to experience physical punishment. Kazakh culture, similar to many other ethnic minority cultures, may have unique child-rearing practices that prioritize non-violent methods of discipline. Research has demonstrated that cultural norms and values significantly impact parenting practices and attitudes toward discipline. For example, Lansford et al. [30] emphasize that the cultural context is crucial in determining how physical punishment is viewed and implemented within families.

Our study focuses on collecting data from mothers or primary caretakers of children under 5 using a structured questionnaire administered through Computer-Assisted Personal Interviewing (CAPI), utilizing tablet computers running Windows 10. In contrast, a 2018 study by Choi et al. [10] conducted direct interviews with children aged 6–17 in Vietnam to investigate physical child punishment, while another study by Kandel et al. [19] carried out household surveys with children aged 10–18 in Nepal to explore child maltreatment, bypassing caregivers. Additionally, a study by Alsarhi et al. in 2019 [31] targeted children aged 6–12 in Yemen, utilizing structured interviews and observations to study maternal harsh physical parenting. Unlike our caregiver-focused approach, these studies directly involve children, providing insights into their experiences and perceptions, with the potential to reveal nuances in child-rearing practices, discipline, and behavioral outcomes. Each study’s methodology offers a unique perspective on child well-being, underscoring the importance of considering both caregiver and child viewpoints in comprehending the complexities of child development and parenting practices.

The diverse methodologies used in previous studies to measure child maltreatment vary. For example, a study on socioeconomic status and physical punishment in Viet Nam used binary coding for physical punishment, inquiring about five specific actions in the past 4 weeks, with any affirmative response indicating punishment [10]. Similarly, the Nepal NMICS study utilized the Parent-Child Conflict Tactics Scale to distinguish between moderate and severe physical abuse and measure emotional abuse, creating binary outcomes from aggregated scores [32]. The Nepal prevalence study defined physical punishment based on specific disciplinary acts in the past month and explored attitudes toward child punishment and intimate partner violence [19]. By discussing these varied methodologies, we aim to illustrate the impact of different thresholds on prevalence estimates. Our use of any affirmative response ensures comprehensive identification of abuse but may include isolated incidents that other studies might not consider maltreatment. Addressing these methodological differences will enhance the robustness of our findings and contribute to the broader discourse on child maltreatment research.

Studies have shown that attitudes toward physical punishment of children vary across cultures [8]. For instance, in Mongolia, a moderate proportion of caregivers endorse physical punishment, while countries like Belarus and Montenegro exhibit more progressive views [8]. Conversely, in places like Burkina Faso and Cameroon, physical punishment is culturally accepted [8]. In Mongolia, nearly 19% of caregivers view physical punishment as necessary [14]. However, a significant portion also supports non-violent disciplinary methods. This highlights the need to understand cultural norms when defining physical and psychological aggression. Just because certain actions may be culturally normalized in some regions does not mean they are not harmful. Recognizing these nuances is crucial for promoting positive parenting practices globally.

To the best of our knowledge, this is the first study in Mongolia to investigate the prevalence of and factors associated with physical punishment and psychological aggression toward children under 5 years of age. Utilizing data from the MICS6, this study benefits from a nationally representative sample and standardized sampling methods, ensuring the robustness and generalizability of the findings. Despite these strengths, this study has some limitations. First, the study’s cross-sectional design limited the inference of causality and the exploration of temporal relationships between variables, emphasizing that the identified associations should be interpreted with caution. The second limitation may be the constraints of previous research and underscores the need for ongoing investigations to deepen our understanding of the complex interplay between sociocultural factors and disciplinary practices.

## Conclusion

Using the 2018 MICS data, the study identified individual (child and household) and regional characteristics of children under 5 years of age in Mongolia. Factors related to physical punishment and psychological aggression in the month before the survey were analyzed. The findings revealed two key risk factors related to child discipline practices: the age of the child (3–4 years old) and the religion of the household head. Buddhist household heads increased the prevalence of psychological aggression.

This study emphasizes the importance of specific policy interventions to address the prevalence of physical punishment and psychological aggression toward children under five in Mongolia. This highlights the necessity for age-sensitive parental education programs, particularly tailored to parents of the 3- to 4-year-old age group, aiming to equip parents with effective, nonviolent discipline strategies aligned with toddlers’ developmental stages. Additionally, the observed association between the religion of the household head and disciplinary practices underscores the call for religious and cultural sensitivity in safeguarding measures, necessitating policies that address the vulnerabilities faced by children in religious settings and establish robust mechanisms for reporting and preventing abuse.

## Appendices Appendix 1. STROBE statement

**Table:**

	Item No	Recommendation	Page No
Title and abstract	1	(*a*) Indicate the study’s design with a commonly used term in the title or the abstract	v
		(*b*) Provide in the abstract an informative and balanced summary of what was done and what was found	vi
Introduction			
Background/rationale	2	Explain the scientific background and rationale for the investigation being reported	1,2
Objectives	3	State specific objectives, including any prespecified hypotheses	3
Methods			
Study design	4	Present key elements of study design early in the paper	4,5
Setting	5	Describe the setting, locations, and relevant dates, including periods of recruitment, exposure, follow-up, and data collection	5
Participants	6	(*a*) Give the eligibility criteria, and the sources and methods of selection of participants	6
Variables	7	Clearly define all outcomes, exposures, predictors, potential confounders, and effect modifiers. Give diagnostic criteria, if applicable	7
Data sources/measurement	8*	For each variable of interest, give sources of data and details of methods of assessment (measurement). Describe comparability of assessment methods if there is more than one group	7,8
Bias	9	Describe any efforts to address potential sources of bias	N/A
Study size	10	Explain how the study size was arrived at	6
Quantitative variables	11	Explain how quantitative variables were handled in the analyses. If applicable, describe which groupings were chosen and why	N/A
Statistical methods	12	(*a*) Describe all statistical methods, including those used to control for confounding	6,7
		(*b*) Describe any methods used to examine subgroups and interactions	N/A
		(*c*) Explain how missing data were addressed	
		(*d*) If applicable, describe analytical methods taking account of sampling strategy	N/A
		(*e*) Describe any sensitivity analyses	N/A
Results			
Participants	13*	(a) Report numbers of individuals at each stage of study – e.g. numbers potentially eligible, examined for eligibility, confirmed eligible, included in the study, completing follow-up, and analyzed	10
		(b) Give reasons for non-participation at each stage	N/A
		(c) Consider use of a flow diagram	N/A
Descriptive data	14*	(a) Give characteristics of study participants (e.g. demographic, clinical, social) and information on exposures and potential confounders	14
		(b) Indicate number of participants with missing data for each variable of interest	N/A
Outcome data	15*	Report numbers of outcome events or summary measures	N/A
Main results	16	(*a*) Give unadjusted estimates and, if applicable, confounder-adjusted estimates and their precision (e.g. 95% confidence interval). Make clear which confounders were adjusted for and why they were included	15,16
		(*b*) Report category boundaries when continuous variables were categorized	N/A
		(*c*) If relevant, consider translating estimates of relative risk into absolute risk for a meaningful time period	N/A
Other analyses	17	Report other analyses done – e.g. analyses of subgroups and interactions, and sensitivity analyses	N/A
Discussion			
Key results	18	Summarize key results with reference to study objectives	17,18
Limitations	19	Discuss limitations of the study, considering sources of potential bias or imprecision. Discuss both direction and magnitude of any potential bias	19
Interpretation	20	Give a cautious overall interpretation of results considering objectives, limitations, multiplicity of analyses, results from similar studies, and other relevant evidence	19
Generalizability	21	Discuss the generalizability (external validity) of the study results	N/A
Other information			
Funding	22	Give the source of funding and the role of the funders for the present study and, if applicable, for the original study on which the present article is based	N/A

## Appendix 2. Definition of terms

### Violence against children

The UN defines violence against children in line with article 19 of the Convention on the Rights of the Child (UNCRC): ‘All forms of physical or mental violence, injury and abuse, neglect or negligent treatment, maltreatment or exploitation, including sexual abuse.’

**Children’s disciple behavior types are classified as follows.**

**Psychological aggression**: Shouting, yelling, or screaming at a child, as well as calling a child offensive name such as ‘dumb’ or ‘lazy.’

**Physical punishment**: Shaking, hitting, or slapping a child on the hand/arm/leg; hitting the bottom or elsewhere on the body with a hard object; spanking or hitting the bottom with a bare hand; hitting or slapping on the face, head, or ears; and hitting or beating hard and repeatedly.

**Severe physical punishment**: Hitting or slapping a child on the face, head, or ears, and repeatedly hitting or beating a child.

**Violent discipline**: Any physical punishment and/or psychological aggression.

**Non-Violent discipline**: Taking away privileges, explaining why the behavior was wrong, giving the child an alternative activity, and not resorting to any of the violent behaviors mentioned above.
